# Usefulness of total bladder capacity and post-void residual urine volume as a predictor of detrusor overactivity with impaired contractility in stroke patients

**DOI:** 10.3892/etm.2012.708

**Published:** 2012-09-14

**Authors:** SANG HYUB LEE, JOONG GEUN LEE, GYEONG EUN MIN, HYUNG-LAE LEE, CHOONG HYUN LEE, KOO HAN YOO

**Affiliations:** Department of Urology, School of Medicine, Kyung Hee University, Seoul 134-727, Republic of Korea

**Keywords:** urodynamic, stroke, neurogenic urinary bladder

## Abstract

Detrusor overactivity (DO) with impaired contractility (DOIC) is a mixed pattern of involuntary contraction and increased post-void residual urine volume (PVR) which occurs in stroke patients. Urodynamic study results obtained from patients with detrusor abnormalities and stroke were analyzed to identify the associations between various urodynamic parameters and DOIC. Between August 2003 and November 2010, 127 patients were selected from 178 patients undergoing urodynamic study due to urinary symptoms. Stroke patients were divided into three groups: the DO, DOIC and detrusor underactivity (DU) groups. The significance of differences between the three groups was analyzed using the Kruskal-Wallis test and receiver operating characteristic (ROC) curves were used to calculate the accuracy of the urodynamic study result factors to distinguish between the three groups. The average total bladder capacity (TBC) was 219.15±98.30 ml in the DO, 330.25±115.75 ml in the DOIC and 486.00±111.48 ml in the DU (P<0.001) group. The average PVR was 22.64±20.85 ml in the DO, 146.87±95.09 ml in the DOIC and 425.33±136.70 ml in the DU (P<0.001) group. A ROC curve of DO and DOIC revealed that TBC and PVR were significantly different between the DOIC and DO groups. The area under the curve (AUC) of TBC was 0.812 (P<0.001) and that of PVR was 0.987 (P<0.001). A ROC curve of DOIC and DU revealed that TBC and PVR were significantly different between the DOIC and DU groups. The AUC of TBC was 0.813 (P<0.001) and that of PVR was 0.929 (P<0.001). In the urodynamic study of stroke patients with urinary symptoms, TBC and PVR may provide useful information for treating patients who cannot undergo urodynamic study.

## Introduction

Stroke is one of the most serious events that affects the elderly population. Stroke patients suffer impairment, including the loss of memory, vision, speech, motor function and voiding control (1). A number of stroke patients suffer from lower urinary tract symptoms (LUTS) that include frequency, urgency, urge incontinence, dysuria and urinary retention (2). Untreated voiding symptoms may have a major negative impact on the quality of life of stroke patients and their relatives and are associated with a high mortality risk (3–5). LUTS are important to urologists and it is essential to evaluate detrusor function to manage LUTS in stroke patients (6).

The majority of common urinary symptoms in stroke patients are storage symptoms, such as nocturia, urgency, frequency and urge incontinence. However, certain patients complain of voiding symptoms such as strain, weak stream and tenesmus (6). Stroke patients may experience storage and voiding symptoms together (7). When these symptoms occur together it is termed detrusor overactivity (DO) with impaired contractility (DOIC). DOIC is complicated to manage and presents a challenging clinical problem (8). Stroke patients with LUTS should undergo urodynamic study to evaluate their detrusor function, particularly when the patient has urgency, urge incontinence, weak stream or tenesmus. However, urodynamic examination of stroke patients is a difficult task due to their poor condition or inability to cooperate.

In the present study, the urodynamic characteristics of patients with detrusor abnormalities and stroke were retrospectively analyzed to ascertain how total bladder capacity (TBC) and post-void residual urine volume (PVR) were reflected in diagnosing DOIC. The aim of the present study was to develop a simple method for detecting DOIC.

## Patients and methods

### Patients

Between August 2003 and November 2010, 178 stroke patients underwent urodynamic study due to LUTS. A total of 51 patients were excluded due to prostate volumes >35 ml on transrectal ultrasonography, poorly controlled diabetes, bladder outlet obstruction, detrusor-external urethra sphincter dyssynergia or normal findings on urodynamic study. Informed consent was obtained from all patients.

### Urodynamic studies

The urodynamic studies were performed using the Libra urodynamic test system (Medical Measurement Systems, Enschede, The Netherlands) (6). All patients were cooperative and free of any urological medications, such as antimuscarinic agents or α-blockers, which may have affected the test. Each patient was supine for a check that the bladder was empty prior to performing the tests. An 8-French double lumen catheter was inserted into the bladder via the urethra to fill the bladder and measure the bladder pressure. The bladder was filled with room temperature sterile saline at a rate of 30 ml/min. Intravesical pressures were checked during all the tests. As saline flushing of the bladder proceeded, each patient was asked if they had a desire to urinate. The points of the patients' first, normal and strong desire to urinate were recorded. Filling of the bladder was stopped when the desire to urinate was strong and represented the cystometric capacity. When filling was complete, the patients were requested to urinate. Urodynamic parameters, including TBC, PVR, voided volume (VV), peak flow rate (Qmax), average flow rate (Qavg), compliance and detrusor pressure at peak flow rate (PdetQmax) were measured.

Patients were divided into three groups according to the results. The detrusor underactivity (DU) group was defined as voiding with abdominal pressure instead of detrusor contraction, residual urine volume >50 ml or an inability to void (9,10). The DO group included patients who exhibited involuntary detrusor contraction during the filling phase (2). The third group was the DOIC group. Since the diagnostic criteria of DOIC have not been confirmed by the International Continence Society (ICS), DOIC was defined as the presence of involuntary contraction during the filling phase and under-active detrusor function during voiding phase, voiding with abdominal pressure only or residual urine >50 ml (2,9,10).

### Statistical analysis

The significance of differences between the three groups was analyzed using the Kruskal-Wallis test. The baseline characteristics and urodynamic parameters of each group were compared. Receiver operating characteristic (ROC) curves were drawn to clarify which parameters best aided in determining the status of DOIC. P<0.05 was considered to indicate a statistically significant result.

## Results

### Patients

Of the 127 patients, 57 were male and 70 were female. The basic characteristics of each group are shown in Table I. The patient numbers of the DO, DOIC and DU groups were 58 (45.6%), 16 (12.5%) and 53 (41.7%), respectively, and the mean ages were 64.13, 67.13 and 67.90 years, in the same respective order. Comparing male and female patients did not reveal any significant differences. Furthermore, there was no correlation between a patient's gender and neurogenic bladder type. No significant differences were observed among the three groups in the mean interval between the stroke event and the test.

### Urodynamic studies

When DOIC was compared with DO, the two groups demonstrated significant differences in TBC and PVR. The urodynamic factors of the two groups are shown in Table II. The average TBCs of the DO and DOIC groups were 219.15±98.30 and 330.25±115.75 ml, respectively. The average PVRs of the DO and DOIC groups were 22.64±20.85 and 146.87±95.09 ml, respectively. The differences between the two groups were significant (P<0.001). However, no significant differences were observed for the other factors. The average VV of each group was 196.67±101.46 (DO) and 185.87±108.48 ml (DOC; P=0.724). The other urodynamic factors, including Qmax, Qavg, compliance and PdetQmax, were not observed to be significantly different between the two groups.

**Figure 1:**
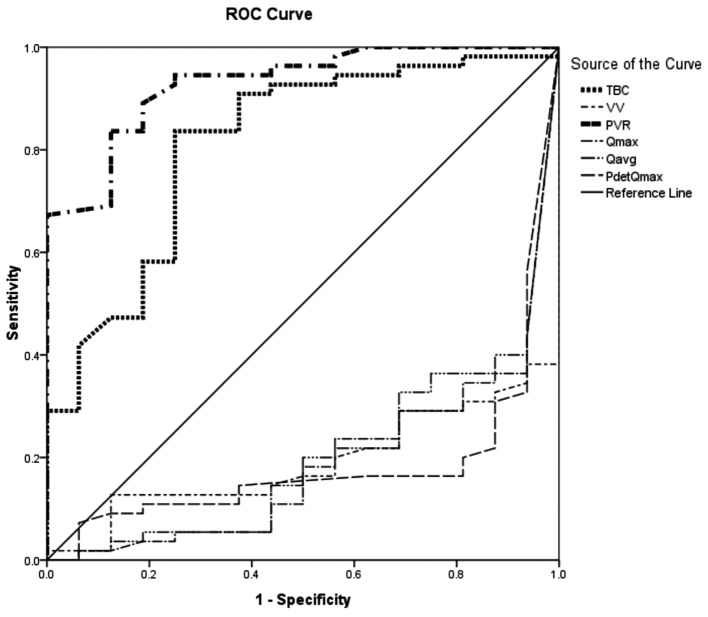
Receiver operating characteristic curve of detrusor overactivity group and detrusor hyperactivity with impaired contractility group. TBC, total bladder capacity; VV, voiding volume; PVR, post-void residual volume; Qmax, peak flow rate; Qavg, average flow rate; PdetQmax, detrusor pressure at peak flow rate.

Similar results were revealed by the ROC curve of DOIC and DO. Fig. 1 shows a ROC curve of DOIC and DO. The area under the curve (AUC) of TBC was 0.812 (P<0.001) and that of PVR was 0.987 (P<0.001). However, the AUC of VV was 0.474 and those of Qmax, Qavg and PdetQmax were 0.421, 0.591 and 0.369, respectively. Only TBC and PVR were significantly different and clinically useful factors for distinguishing DOIC from DO. Compared with the DO group, if the cut-off value of TBC in the DOIC group was set to >262.50 ml, the sensitivity was 90.9% and the specificity was 68.4%. If the cut-off value of PVR in the DOIC group was set to >67.50 ml then the sensitivity was 90.9% and the specificity was 94.7%.

By contrast, the DOIC and DU groups demonstrated significant differences in all urodynamic factors. The factors of the two groups are presented in Table III. The average TBCs of the DOIC and DU groups were 330.25±115.75 and 486.00±111.48 ml (P<0.001), respectively. The average PVRs of the DOIC and DU groups were 146.87±95.09 and 425.33±136.70 ml, respectively, and the difference between the two groups was significant (P<0.001). The average VVs of the two groups were 185.87±108.48 (DOIC) and 63.94±109.30 ml (DU) and were significantly different (P= 0.001). The other urodynamic factors, including Qmax, Qavg, compliance and PdetQmax also exhibited significant differences between the two groups.

**Figure 2:**
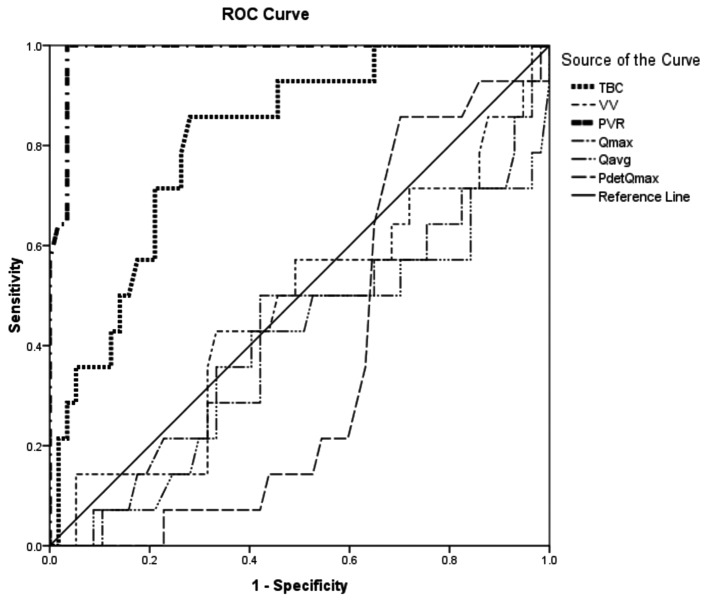
Receiver operating characteristic curve of detrusor hyperactivity with impaired contractility group and detrusor overactivity group. TBC, total bladder capacity; VV, voiding volume; PVR, post-void residual volume; Qmax, peak flow rate; Qavg, average flow rate; PdetQmax, detrusor pressure at peak flow rate.

Fig. 2 presents a ROC curve of DOIC and DU. TBC and PVR were significantly different between the DOIC and DU groups. The AUC of TBC was 0.813 (P<0.001) and that of PVR was 0.929 (P<0.001). The cut-off value was also determined. Compared with the DU group, if the cut-off value of TBC in the DOIC group was set to <335 ml, the sensitivity was 90.3% and the specificity was 72.7%. If the cut-off value of PVR in the DOIC group was set to <220 ml then the sensitivity was 90.3% and the specificity was 81.8%.

TBC and PVR were the only factors that were able to distinguish DOIC from DO and DU. The TBC and PVR of the DOIC group were greater than those of the DO group but smaller than those of the DU group. Therefore, the ranges of TBC and PVR were useful for diagnosing DOIC using the ROC curves. If the TBC and PVR of patients with LUTS were 262.5–335 and 67.5–220 ml, respectively, DOIC may be predicted.

## Discussion

Since the first description of patients with DOIC, numerous studies concerning DOIC have been published (8,11). The reported criteria for DOIC are a combination of urodynamically proven urgency or urge incontinence and a high PVR. In the present study, DOIC was defined more strictly urodynamically in terms of using abdominal pressure instead of detrusor contraction in voiding or a PVR of over 50 ml, indicating impaired contractile function (9,10). The present urodynamic criteria for DOIC yielded clinical results that did not differ from those of previous studies (8,11).

The high prevalence of LUTS, including urinary incontinence, in stroke patients has been reported previously (12,13). Incontinence may persist for months following the event (3). Stroke patients with urinary incontinence have a higher risk of mortality than stroke patients who are not incontinent. Urinary incontinence in stroke patients has a significant impact on their lives, frequently including greater morbidity and mortality (14,15). However, the previously mentioned studies noted that not only urinary incontinence but also other urinary tract symptoms, such as nocturia, frequency, urgency, pain and straining affect quality of life. Two studies reported all the possible urinary symptoms of stroke patients (14,15). These studies focused not only on urge incontinence but also frequency, nocturia and straining (3,14,15). Tibaek *et al* reported that at least one LUTS may bother stroke patients (16). In the present study, the most frequent symptom in females and males was nocturia (76% for both) followed by urgency (70 and 61%, respectively). Furthermore, 60% of all patients (51% females, 67% males) suffered from voiding symptoms such as weak stream, hesitancy, incomplete emptying or straining.

Few studies have mentioned the association between stroke and DOIC. Yamamoto *et al* suggested that numerous neurological diseases cause DOIC and that multiple cerebral infarction (MCI) is one cause of DOIC (7). The authors reported that 9 of 70 (12%) MCI patients had DOIC. However, the authors noted that the study did not consider and examine a combination of multiple diseases, such as MCI and diabetic cystopathy. Tibaek *et al* reported that stroke may affect the patients' voiding symptoms, precluding the development of a variety of LUTS (16). The authors surveyed 482 stroke patients and noted both storage and voiding symptoms in 43% of this population. Although the authors did not perform a urodynamic study and instead gathered information solely by a questionnaire, it appears likely that at least some of these patients with both symptoms had DOIC. The design of the present study enabled the determination of the presence of storage and voiding symptoms in DOIC patients.

Previously, we were able to evaluate the bladder function of stroke patients by the type of stroke (6) and concluded that an evaluation of the stroke type is likely to aid the determination of the type of urinary dysfunction and in deciding the appropriate bladder management. However, we observed no significant differences in LUTS between dominant, non-dominant and bilateral hemispheric ischemic stroke patients (17). It has been argued that despite problems in elderly patients, it is reasonable to perform complex urodynamic studies (18). The collective results favor the suggestion that urodynamic examination is necessary for stroke patients with LUTS to understand detrusor function and manage voiding symptoms.

Diagnosis and management of DOIC are difficult. Empirical prescription of anticholinergic agents may lead to acute urinary retention and α-blockers may aggravate incontinent urgency (8). To manage DOIC, precise knowledge of detrusor function is necessary. The common criteria for DOIC are involuntary contraction during the filling phase and no detrusor contraction during the voiding phase or a high PVR. Studies that have urodynamically defined DOIC have concluded that this strategy is necessary for a diagnosis of DOIC. However, this strategy is challenging for stroke patients with DOIC who cannot cooperate with the examiner in expressing their intention or whose urine analysis reveals consistent pyuria. Problems unique to the evaluation of elderly patients with stroke include technical difficulties in interpreting urodynamic results (8). Consequently, if there was an easy method for predicting DOIC, clinicians may choose this method for stroke patients whose cooperation is difficult to achieve.

In the present study, the DU, DOIC and DO groups revealed significant differences in all urodynamic factors which may reflect different characteristics of the groups. In ROC curve analysis, TBC and PVR were significant factors for distinguishing DOIC from DU or DO. Checking TBC and PVR may aid clinicians in the diagnosis of DOIC. By determining the cut-off value, DOIC may be predicted when TBC is 262.5–335 ml and PVR is 67.5–220 ml.

To clarify the criteria and definition of DOIC, further functional studies are required. Since there is no easy method to diagnose DOIC, urodynamic examination is an inevitable test for patients with storage and emptying symptoms. However, in patients for whom urodynamic information cannot be easily obtained, the evaluation of TBC and PVR may be useful for the determination of DOIC and bladder management. TBC and PVR data may be used to predict whether stroke patients in poor condition have DOIC.

In conclusion, evaluation of TBC and PVR may be useful for diagnosing DOIC in stroke patients who cannot undergo urodynamic study. DOIC may be predicted when TBC and PVR are within target ranges.

## Figures and Tables

**Table I:** Baseline characteristics of study population.

Characteristic	DO	DOIC	DU	P-value
Number of patients (%)	58 (45.6)	16 (12.5)	53 (41.7)	
Age (years), mean (range)	64.13 (23–87)	67.31 (51–79)	67.90 (45–83)	0.420
Interval (months), mean (±SD)	13.95±27.84	9.31±16.03	21.32±47.30	0.506

DO, detrusor overactivity; DOIC, detrusor overactivity with impaired contractility; DU, detrusor underactivity. There were 127 patients, of whom 57 were male and 70 were female.

**Table II:** Urodynamic factors of the DO and DOIC groups. TBC and PVR were the only factors which had significant differences between the two groups.

Factor	DO, mean ± SD	DOIC, mean ± SD	P-value
TBC (ml)	219.15±98.30	330.25±115.75	0.002
VV (ml)	196.67±101.46	185.87±108.48	0.724
PVR (ml)	22.64±20.85	146.87±95.09	<0.001
Qmax (ml/sec)	15.12±9.98	11.95±6.66	0.152
Qavg (ml/sec)	6.74±3.80	5.28±3.19	0.142
Compliance	25.18±72.43	16.98±23.46	0.498
PdetQmax (ml/sec)	41.05±24.12	30.00±10.59	0.011

DO, detrusor overactivity; DOIC, detrusor overactivity with impaired contractility; TBC, total bladder capacity; VV, voiding volume; PVR, post-void residual volume; Qmax, peak flow rate; Qavg, average flow rate; PdetQmax, detrusor pressure at peak flow rate.

**Table III:** Urodynamic factors of the DU and DOIC groups. All urodynamic factors had significant differences between the two groups.

Factor	DU, mean ± SD	DOIC, mean ± SD	P-value
TBC (ml)	486.00±111.48	330.25±115.75	<0.001
VV (ml)	63.94±109.30	185.87±108.48	0.001
PVR (ml)	425.33±136.70	146.87±95.09	<0.001
Qmax (ml/sec)	3.43±5.23	11.95±6.66	0.003
Qavg (ml/sec)	1.66±2.51	5.28±3.19	0.010
Compliance	139.04±206.60	16.98±23.46	0.002
PdetQmax (ml/sec)	10.56±15.07	30.00±10.59	0.001

DOIC, detrusor overactivity with impaired contractility; DU, detrusor underactivity; TBC, total bladder capacity; VV, voiding volume; PVR, post-void residual volume; Qmax, peak flow rate; Qavg, average flow rate; PdetQmax, detrusor pressure at peak flow rate.
